# Supramolecular Luminescence from Oligofluorenol-Based Supramolecular Polymer Semiconductors

**DOI:** 10.3390/ijms141122368

**Published:** 2013-11-13

**Authors:** Guang-Wei Zhang, Long Wang, Ling-Hai Xie, Jin-Yi Lin, Wei Huang

**Affiliations:** 1 Center for Molecular Systems and Organic Devices (CMSOD), Key Laboratory for Organic Electronics & Information Displays, Institute of Advanced Materials, Nanjing University of Posts & Telecommunications, Nanjing 210046, China; E-Mails: iamgwzhang@njupt.edu.cn (G.-W.Z.); mrlong8@126.com (L.W.); y007091815@njupt.edu.cn (J.-Y.L.); 2 Jiangsu-Singapore Joint Research Center for Organic/Bio-Electronics & Information Displays, Institute of Advanced Materials, Nanjing University of Technology, Nanjing 211816, China

**Keywords:** luminescence, supramolecular polymers, polyfluorenes, hydrogen bonds, thin films

## Abstract

Supramolecular luminescence stems from non-covalent exciton behaviors of active π-segments in supramolecular entities or aggregates via intermolecular forces. Herein, a π-conjugated oligofluorenol, containing self-complementary double hydrogen bonds, was synthesized using Suzuki coupling as a supramolecular semiconductor. Terfluorenol-based random supramolecular polymers were confirmed via concentration-dependent nuclear magnetic resonance (NMR) and dynamic light scattering (DLS). The photoluminescent spectra of the TFOH-1 solution exhibit a green emission band (g-band) at approximately ~520 nm with reversible features, as confirmed through titration experiments. Supramolecular luminescence of TFOH-1 thin films serves as robust evidence for the aggregates of g-band. Our results suggest that the presence of polyfluorene ketone defects is a sufficient condition, rather than a sufficient-necessary condition for the g-band. Supramolecular electroluminescence will push organic devices into the fields of supramolecular optoelectronics, spintronics, and mechatronics.

## 1. Introduction

Carbon is the smartest element in the periodic table. In this century, organic/plastic electronics are impacting the human society through organic light-emitting devices (OLEDs), solar cells, thin film transistors, organic lasers, organic memory devices and so on, which have all attracted significant attention from not only scientists but also the industrial community. Smart organic devices will update complex organic matter and function into organic mechatronics that involves mechano-semiconductors, molecular consciousness and organic robotics in the coming era of consciousness ultimately. Over the last few decades, powerful organic syntheses and rational top-down molecular design have enabled significant progress in organic electronics and OLED that have been commercially available [1]. Up to date, a bottom-up chemical four-element theory that involves electronic structures, steric repulsions, conformational topology, and non-covalent attractions, has been extracted to guide the molecular design of organic/polymer semiconductors [2]. However, several big challenges remain in terms of low-cost thin film deposition procedures. Relative to energy-consumptive vacuum thermal evaporation technique, solution-processed organic devices face the challenges such as the uniform, repeatable and multilayer high-quality thin films owing to unpredictable molecular packing, hierarchical textiles, and complex condensed behaviors.

Noncovalent forces dominate the condensed behaviors of solution-processed organic thin films. Supramolecular analyses in organic/polymer semiconductors have become an complementary top-level tool for understanding organic devices, especially for solution-processable ones. Supramolecular approaches enables the tuning of the electronic structures and energy transfers effectively. Supramolecular functionalization that introduces diverse noncovalent moieties to π-conjugated organic/polymer semiconductors via the covalent linkages imparts appealing features such as soft-matter mechanical features, self-assembly, self-healing and stimulus-response behaviors, besides the intrinsic optoelectronic characters. Significant progress has been made in terns of mainchain-type supramolecular polymers via multiple hydrogen bonds to understand complex pathway of supramolecular polymerizations [3]. However, the interruption of their conjugation sometimes limit the electronic delocalization and worsen electron transport. The π-stacked polymers was coined as supramolecular organic semiconductors (SOSs) that have the unique supramolecular channels for the charge transfer of electrons and generation of exciton [4–6]. These supramolecular conductive channels exhibit the unique dynamics, reversible conformations, and stimulus-response features that can be adjusted and controlled via bulky groups. As a result, hindrance-functionalized stacked polymers exhibit a conformational switching feature with the potential application of resistive nonvolatile flash memories. Supramolecular π-conjugated polymers are alternative side-chain-type supramolecular polymer semiconductors (SPSs) [7], with the obvious advantages of both excellent electronic transport and molecular assembly, which opens the door to smart organic devices in the fields of supramolecular optoelectronic and mechatronics.

Of the various supramolecular forces, hydrogen bonding is a pivotal role to bio-systems, supramolecular assemblies, and organic semiconductors. Various multiple hydrogen bonds have been designed and synthesized [8–14] via supramolecular chemistry. Our previous works, demonstrate the supramolecular functionalization of polyfluorene-based conjugated polymers via hydrogen bonding, to design poly(tertiary alcohol) as excellent gelators [15]. As a result, gelation becomes a concise standard, guiding the selection of solvents in organic optical inks, which are key component to spin-coating or inkjet printing techniques. Our results also show that non-covalent network precursors in the solution are crucial to the thin-film optoelectronic behaviors and morphologies. These results inspired us to explore the supramolecular luminescence that stems from supramolecular structures or aggregates, consisting of intermolecular active π-segments, which are reorganized via non-covalent forces. In addition, the green emission bands (g-band) of polyfluorenol have been transferred as g-bands in polyfluorene and are crucial to the stability and performance of blue polymer light-emitting diodes (PLEDs), which has hindered their commercialization [16]. By comparison to polymer semiconductors, low molecular weight organic molecules have advantages over polymers in terms of the their structure-property relationships [17,18]. In this article, we design oligomers to confirm that the polyfluorenol g-bands stem from superstructures of the backbone, rather than chemical ketone defects. Our results differ completely from a previous study of a terfluorene with a flurorenol in the middle position [19].

## 2. Results and Discussion

### 2.1. Synthesis

**Scheme 1:**
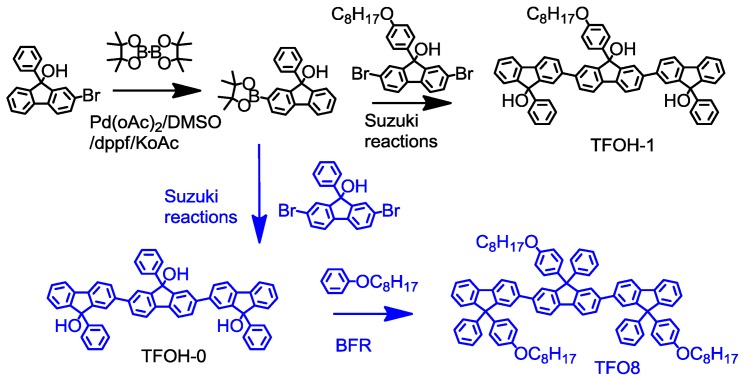
Synthetic routes to TFOH-1 and the TFO8 control.

To obtain supramolecular luminescence and clarify the green emission bands of polyfluorenols, we used terfluorenol TFOH-1 (9′-(4-(octyloxy)phenyl)-9,9″-diphenyl-[2,2′:7′,2″-terfluorene]-9,9′,9″-triol) as model supramolecular semiconductors and a bulky ter(diphylfluorene) TFO8 (9,9′,9″-tris (4-(octyloxy)phenyl)-9,9′,9″-triphenyl-2,2′:7′,2″-terfluorene), as the sterically hindered reference sample. The synthetic routes for these compounds are shown in Scheme 1. The fluorene tertiary alcohol monomer was synthesized via a Grignard reaction as previously reported [20]. The target oligofluorenol were synthesized via a palladium-catalyzed carbon-carbon Suzuki cross-coupling reaction with approximately 75% yield due to its tolerance to hydroxyl groups. The TFO8 control, which has no supramolecular hydrogen bonding, was synthesized via a BF_3_·OEt_2_-mediated Friedel-Crafts reaction (BFR) [20] between oligofluorenol and (octyloxy)benzene with a yield of up to 90%.

### 2.2. Supramolecular Polymers and Aggregates in Solutions

**Figure 1:**
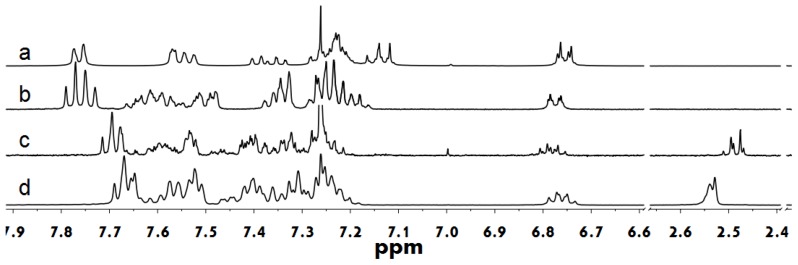
^1^H NMR spectra of TFO8 in CDCl_3_ (**a**); ^1^H NMR spectra of TFOH-1 in different solutions; (**b**) CD_3_OD and (**c**,**d**) CDCl_3_ and concentrations; (**b**,**c**) 2 mg/mL and (**d**) 10 mg/mL.

To gain insight into the supramolecular interactions of the designed tertiary alcohol, concentration-dependent nuclear magnetic resonance (NMR) studies were performed in different solvents. Figure 1 shows the ^1^H NMR spectra of the TFO8 control in CDCl_3_ (a), the solvent-dependent ^1^H NMR spectra of 2 mg/mL TFOH-1 in both CD_3_OD (b) and in CDCl_3_ (c) and the concentration-dependent ^1^H NMR spectra of TFOH-1 (c: 2 mg/mL; d: 10 mg/mL). TFO8 yields well-resolved proton signals. The NMR spectra for TFOH-1 in CD_3_OD yielded well-resolved proton signals with no obvious up-field shift relative to TFO8, which indicates there are weak π–π stacking interactions between backbone chains owing to CD_3_OD-TFOH-1 hydrogen bonding complexes. Conversely, the aromatic proton signals for TFOH-1 in CDCl_3_ (c–d) were shifted (Δδ ≈ −0.08 ppm), relative to the aromatic protons in (a) and (b), which was attributed to the molecular packing of terfluorene due to interchain hydrogen bonding interactions [21,22]. In the NMR of the concentrated 10 mg/mL solution, the hydroxy hydrogens were shifted downfield (Δδ ≈ +0.06 ppm), which suggests the presence of double hydrogen bonds. Moreover, all of the signals broadened at high concentrations, which indicates supramolecular aggregates or non-covalent, random single-component supramolecular polymers with high molecular weight were present [23]. It was deduced that the hydrogen bonding and π–π stacking interactions most likely dominated the aggregate behaviors for the TFOH-1 in solutions.

**Figure 2:**
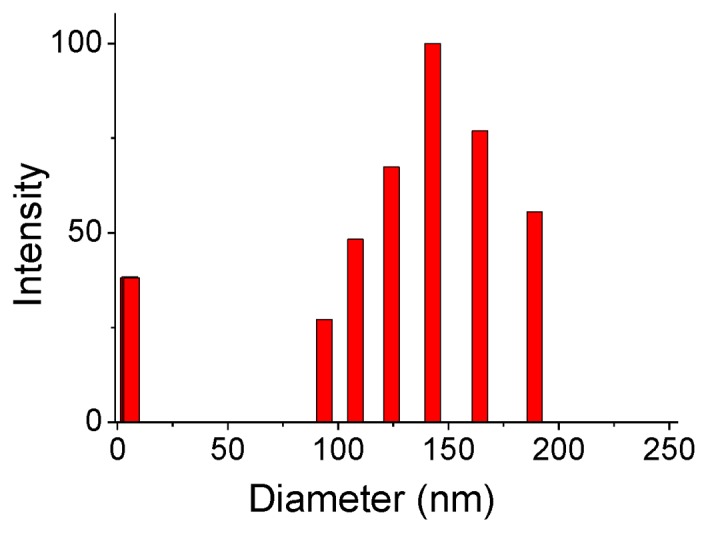
Distribution of hydrodynamic radii (*R*_h_) for the TFOH-1 aggregates (chloroform, 10 mg/mL).

Dynamic light scattering (DLS) was employed to probe the molecular behaviors of TFOH-1 aggregates in chloroform at a concentration of 10 mg/mL (Figure 2). TFOH-1 possessed two sets of hydrodynamic radii. The first set is a narrow distribution of particles 2 to 9 nm in size that corresponds to the monomer and dimer structures. The second sets range from 90 to 190 nm and most likely arise from multiple associations between monomers to yield supramolecular aggregates. Hydroxy-based assemblies are consistent to previous observations [15].

**Figure 3:**
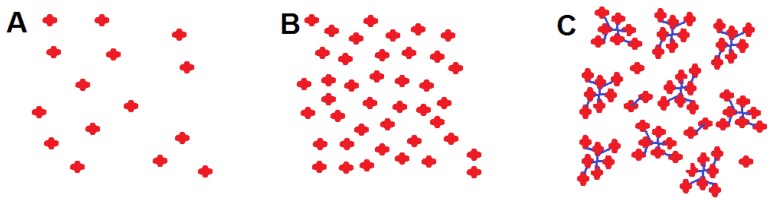
Schematic illustration of the supramolecular TFOH-1 aggregate (red cross: TFOH-1 molecules; blue line: H-bonding). (**A**) Dilute TFOH-1 molecules in chloroform; (**B**) Concentrated TFOH-1 in the CH_3_OH; (**C**) Concentrated TFOH-1 in an aprotic solvent.

According to the NMR and DLS, TFOH-1 exhibited different molecular aggregates in various states as outlined in Figure 3. Dilute TFOH-1 molecules in chloroform are dispersed individually as depicted in Figure 3A. Concentrated TFOH-1 in the CH_3_OH has no interactions between the terfluorene molecular, as shown in Figure 3B, due to the formation of solvent-TFOH-1 complexes that are only expected to slightly change the conjugation length. However, concentrated TFOH-1 in an aprotic solvent, such as chloroform, aggregates via intermolecular hydrogen bonds to form new supramolecular entities as shown in Figure 3C. Therefore, random non-covalent, single-component supramolecular polymers are thought to form an elastic cross-linked network of terfluorenes at room temperature. In this scenario, the optoelectronic behavior of TFOH-1 can be modified via the solvent, solvent concentration, and other external additives or conditions.

### 2.3. Supramolecular Luminescence in Solution and Thin Films

**Figure 4:**
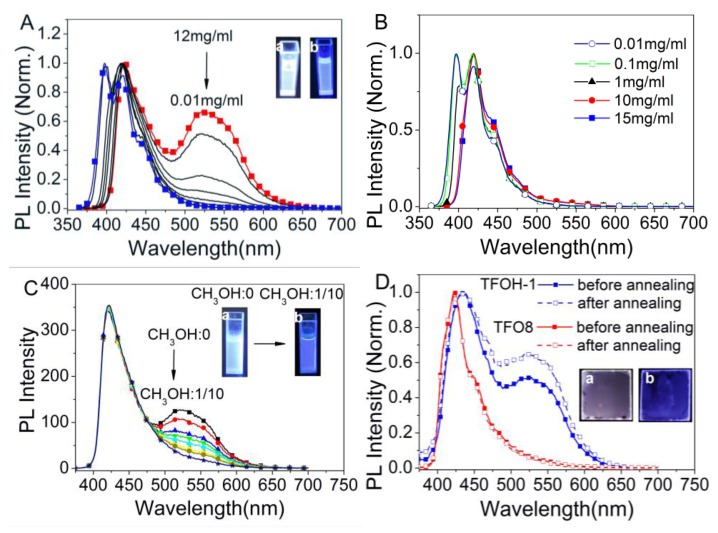
Photoluminescence spectra. (**A**) Concentration dependent PL spectra of TFOH-1 in toluene (0.01–12 mg/mL) with photographs (a for 12 mg/mL; b for 0.01 mg/mL); (**B**) Concentration dependent PL spectra of TFO8 in toluene (0.01–15 mg/mL); (**C**) PL spectra of TFOH-1 in toluene (8 mg/mL) with differing methanol concentrations (0%–10%) with photographs (a for methanol = 0; b for methanol = 10%); (**D**) PL spectra of the films before and after annealing and photographs of thin films after annealing (a for TFOH-1; b for TFO8).

In this supramolecular framework, TFOH-1 photoluminescence spectra and TFO8 control were systemically characterized in different solution and thin films as shown in Figure 4. Based on the NMR and DLS results discussed above, the TFOH-1 supramolecular polymer aggregates that form at high concentrations, result from intermolecular hydrogen bonding. The concentration-dependent PL spectra of TFOH-1 and TFO8 are shown in Figure 4A,B. Dilute TFOH-1 in toluene yields emission peaks at 398 and 420 nm with a shoulder at 448 nm that was attributed to the main terfluorene chains [24], similarly, the dilute TFO8 solution yields peaks at 397 and 419 nm with a shoulder at 443 nm. However, the PL spectra of concentrated TFOH-1 in toluene only possessed two emission peaks at 423 and 524 nm, which was an obvious difference different from the TFO8 solution with the same concentration. No green band emissions (g-bands) were detected in the concentrated TFO8, even at concentrations of up to 15 mg/mL. Increasing the concentration (>0.01 mg/mL) quenches the high band emission at approximately 398 nm and leaves only the emission peak at approximately 420 nm. Additionally, the emission peak at 423 nm did not shift after increasing the concentration to 12 mg/mL. The unique emission behavior of TFOH-1 was most likely caused by an energy transfer from isolated terfluorenes to the supramolecular aggregates via hydrogen bond interactions. These results are consistent with the above NMR spectra and support the conclusion that the average *R*_h_ of 18–41 nm from the DLS analysis resulted from hydrogen bonded aggregates. TFO8 possessed a stable blue emission due to the excellent steric hindrance from bulky diarylfluorenes [17].

The supramolecular luminescence behavior of concentrated TFOH-1 in toluene was supported by a titration experiment that involved adding differing CH_3_OH concentrations (0%–10%) to a TFOH-1 in toluene solution (8 mg/mL) [21]. In contrast to TFOH-1 in toluene without methanol, the TFOH-1 emission spectra after adding 10% CH_3_OH exhibited only one peak at 422 nm, which indicates that the terfluorenes formed a well-dissolved isotropic phase corresponding to the NMR analysis. The reversibility of the luminescent supramolecular g-bands in solution strongly supports an aggregate mechanism over the ketone defects mechanism supported by many groups [19,25,26]. The PL spectra of TFOH-1 and TFO8 films spin-coated from toluene solvent (10 mg/mL) were investigated before and after annealing (Figure 4D). Emission spectrum of TFO8 contained a single peak at 435 nm with a shoulder peak at 450 nm. However, similar to its solution emission spectra, the PL spectrum of the TFOH-1 film contained two peaks at 435 nm, the high energy regime associated with single chain emissions, and at 526 nm, which was attributed to TFOH-1 hydrogen bond aggregates. This phenomenon increased significantly after annealing. The supramolecular green emissions supported the aggregate mechanism [27–29] reported by the Tagawa group [30] and our previous results [31]. It is thought that self-constructed supramolecular structures are stable in the spin-coated thin films. These observations all imply the energy transfer from the high-energy regime (individual TFOH-1 chains luminophores in the isotropic phase) to a low-energy entities (intermolecular hydrogen bonded supramolecular polymers) to form an organic bulk homojunction thin film with potential organic electronics applications.

## 3. Experimental Section

### 3.1. Chemicals

All of the solvents and reagents were purchased from commercial suppliers and used without further purification unless otherwise noted. All products were purified by flash column chromatography using Kanto Silica Gel (Kanto Chemical Co., Inc., Xuhui District, Shanghai, China) 60N (40–63 μm). Spectrochemical-grade solvents were used for the optical measurements. Palladium(II) acetate, tetra(triphenylphosphine)palladium(II), 1,1′-bis(diphenylphosphino)ferrocene (dppf), 2,7-dibromo-9-fluorenone, 2-bromo-9-fluorenone, bromobenzene, 1-bromo-4-(octyloxy) benzene, and 2,2′-bithiophene were obtained from Aldrich Chemical Co. (Xuhui District, Shanghai, China). Borontrifluoride etherate, potassium carbonate, magnesium sulfate, chloroform and toluene were purchased from Sinopharm Chemical Reagent Co, Ltd (Nanjing, Jiangsu, China) and were used without further purification. Dichloromethane was dried using anhydrous sodium at room temperature. THF and toluene were dried over the sodium benzophenone ketyl anion radical and distilled under a dry nitrogen atmosphere immediately prior to use. The following 2,7-Dibromo-9- (4-(octyloxy)phenyl)-fluoren-9-ol (DBrOOPFOH), 2,7-dibromo-9-phenyl-fluoren-9-ol (DBrPFOH), 2-bromo-9-phenyl-fluoren-9-ol (BrPFOH) and, 2-(4,4,5,5-tetramethyl-1,3,2-dioxaborolan-2-yl)-9-phenylfluoren-9-ol (TMB-PFOH) were obtained according to previously published protocols [15,25].

### 3.2. Characterization

The ^1^H NMR and ^13^C NMR spectra were recorded in *d*-CDCl_3_ or *d*-CD_3_OD containing a tetramethylsilane (TMS) interval standard, using a Bruker 400 MHz spectrometer. The mass spectra were recorded on a Shimadzu GC-MS 2010 PLUS. The MALDI-TOF MS spectra were recorded in the reflective mode using substrates. The absorption spectra were measured using a Shimadzu UV-3150 spectrometer (Shimadzu Corporation, Nanjing, Jiangsu, China) at 25 °C, and the emission spectra were recorded using a Shimadzu RF-530XPC luminescence spectrometer (Shimadzu Corporation, Nanjing, Jiangsu, China) after excitation at the absorption maxima. Dynamic light scattering (DLS, Zetasizer Nano-ZS) measurements were performed at a wavelength of 633 nm using a laser light source at room temperature. The DLS measurements were generally repeated at least three times, and the average values were reported.

### 3.3. Synthesis

#### 3.3.1. Synthesis of 2-(4,4,5,5-tetramethyl-1,3,2-dioxaborolan-2-yl)-9-phenylfluoren-9-ol (TMB-PFOH) from 2-bromo-9-phenylfluoren-9-ol (BrPFOH)

2-Bromo-9-phenylfluoren-9-ol (3.36 g, 10 mmol, 1 eq), 4,4,4′,4′,5,5,5′,5′-octamethyl-2,2′-bi(1,3,2-dioxaborolane) (3.04 g, 12 mmol, 1.2 eq), palladium(II) acetate (66 mg, 0.3 mmol, 0.03 eq), potassium acetate (2.94 g, 30 mmol, 3 eq), a 1,1′-bis(diphenylphosphino)ferrocene (dppf) ligand (0.332 g, 0.6 mmol, 0.06 eq) were added to a three-necked Schlenk flask (150 mL). The flask was evacuated and back-filled with nitrogen more than three times before injecting with DMSO (30 mL) through a syringe. The mixture was heated to 80 °C and stirred for 4 h. Water (150 mL) was then added to quench the reaction. The phases were separated, and the aqueous phase was extracted with dichloromethane. The combined dichloromethane layers were washed and dried (MgSO_4_). After removing the solvent, the remaining crude product was purified by silicon gel chromatography (petroleum ether-dichloromethane) to afford a pale yellow powder (3.0 g, 79%). GC-MS (*m*/*z*): calcd. For C_25_H_25_BO_3_: 384.2 [M^+^]; Found: 384. ^1^H NMR (400 MHz, CDCl_3_) δ 7.84 (dd, *J* = 7.5, 0.8 Hz, 1H), 7.77 (s, 1H), 7.69 (t, *J* = 7.1 Hz, 2H), 7.41–7.36 (m, 3H), 7.36 (d, *J* = 1.3 Hz, 1H), 7.33 (s, 1H), 7.31 (s, 1H), 7.29 (q, *J* = 2.3 Hz, 1H), 7.24 (dt, *J* = 5.8, 2.0 Hz, 2H), 7.21 (d, *J* = 3.6 Hz, 1H), 2.47 (s, 1H), 1.31 (s, 13H). ^13^C NMR (101 MHz, CDCl_3_) δ 149.73, 142.61, 139.33, 136.02, 129.07, 128.98, 128.22, 127.13, 125.52, 124.85, 120.52, 119.47, 83.86, 83.63, 25.03, 24.99, 24.75 (Figure S1).

#### 3.3.2. Synthesis of TFOH-1 via Suzuki coupling reaction

A typical preparation procedure is as follows: 2,7-Dibromo-9-(4-(octyloxy)phenyl)-fluoren-9-ol (0.54 g, 1 mmol, 1 eq), 9-phenyl-2-(4,4,5,5-tetramethyl-1,3,2-dioxaborolan-2-yl)-fluoren-9-ol (TMB-PFOH) (0.84 g, 2.4 mmol, 2.4 eq), tetra(triphenylphosphine) palladium (0) (35 mg, 0.03 eq) were added to a three-necked Schlenk flask (150 mL). The flask was evacuated and back-filled with nitrogen atmosphere more than three times before injecting with degassed toluene (30 mL) and an aqueous K_2_CO_3_ solution (2 M, 5 mL, 10 eq) through a syringe. The mixture was heated to 90 °C and stirred for 48 h. The solvent was removed under vacuum. The mixture was purified by silica gel chromatography (CH_2_Cl_2_) to afford a pale yellow powder (0.67 g, 75%). MALDI-TOF-MS (*m*/*z*): calcd. For C_65_H_54_O_4_: 898.4 [M^+^]; Found: 896.9 (Figure S2). ^1^H NMR (400 MHz, CDCl_3_) δ 7.67 (dd, *J* = 11.3, 5.5 Hz, 7H), 7.60–7.55 (m, 4H), 7.52 (t, *J* = 5.2 Hz, 5H), 7.48–7.43 (m, 1H), 7.72–7.14 (m, 39H), 7.39 (dd, *J* = 12.7, 7.3 Hz, 5H), 7.31 (ddd, *J* = 12.2, 10.6, 5.8 Hz, 7H), 7.29–7.18 (m, 10H), 6.76 (dd, *J* = 15.1, 6.5 Hz, 2H), 3.95–3.77 (m, 2H), 2.53 (d, *J* = 4.2 Hz, 3H), 1.71 (dt, *J* = 14.5, 7.1 Hz, 2H), 1.46–1.21 (m, 13H), 0.87 (dd, *J* = 7.2, 3.6 Hz, 3H). ^13^C NMR (101 MHz, CDCl_3_) δ 158.45, 151.65, 151.25, 151.18, 150.83, 150.78, 143.09, 143.04, 141.25, 141.19, 141.05, 139.17, 139.11, 138.96, 138.89, 138.34, 132.15, 132.05, 131.91, 129.14, 128.55, 128.47, 128.43, 128.31, 128.27, 128.16, 128.03, 127.27, 126.61, 125.44, 124.78, 123.34, 123.25, 120.43, 120.39, 120.17, 114.29, 83.66, 83.63, 83.43, 67.94, 31.81, 29.35, 29.27, 29.23, 26.07, 26.05, 22.65, 14.10. Anal. calcd. For C_65_H_54_O_4_: C, 86.83; H, 6.05; O, 7.12. Found: C, 87.01; H, 6.71; O, 7.01 (Figure S3).

#### 3.3.3. Synthesis of 9,9′,9″-triphenyl-[2,2′:7′,2″-terfluorene]-9,9′,9″-triol (TFOH-0)

5,5′-Bis(2,7-dibromo-9-(4-(octyloxy)phenyl)-fluoren-9-yl)-2,2′-bithiophene (1.214 g, 1 mmol, 1 eq), 2-bromo-9-(4-(octyloxy)phenyl)-fluoren-9-ol (2.04 g, 4.4 mmol, 4.4 eq), tetra(triphenylphosphine) palladium (0) (35 mg, 0.03 eq) were added to a three-necked Schlenk flask (150 mL). The flask was evacuated and back-filled with nitrogen atmosphere at least three times before injecting with degassed toluene (30 mL) and aqueous K_2_CO_3_ (2 M, 5 mL, 10 eq) through a syringe. The mixture was heated to 90 °C and stirred for two days. The solvent was removed under vacuum. The mixture was purified by silica gel chromatography (CH_2_Cl_2_) to afford a pale yellow powder (0.99 g, 61%). Rf = 0.24. MALDI-TOF-MS (*m*/*z*): calcd. For C_57_H_38_O_3_: 770 [M^+^]; Found: 768.1. ^1^H NMR (400 MHz, CDCl_3_) δ 7.67 (t, *J* = 6.5 Hz, 6H), 7.57 (t, *J* = 7.9 Hz, 4H), 7.52 (t, *J* = 4.9 Hz, 4H), 7.39 (dd, *J* = 13.5, 7.1 Hz, 8H), 7.31 (t, *J* = 7.2 Hz, 3H), 7.24 (dt, *J* = 17.9, 8.1 Hz, 12H), 2.54 (d, *J* = 18.5 Hz, 3H). ^13^C NMR (101 MHz, CDCl_3_) δ 151.24, 151.17, 150.78, 150.74, 143.03, 142.98, 141.26, 141.21, 141.12, 141.07, 139.15, 139.10, 138.99, 138.93, 138.46, 129.17, 128.50, 128.37, 128.32, 128.28, 128.23, 128.17, 128.11, 128.04, 127.29, 125.43, 124.78, 123.35, 123.31, 120.49, 120.42, 120.19, 83.67, 83.63.

#### 3.3.4. Synthesis of 9,9′,9″-tris(4-(octyloxy)phenyl)-9,9′,9″-triphenyl-2,2′:7′,2″-terfluorene (TFO8)

A BF_3_·OEt_2_ complex (0.06 mL, 0.5 mmol) solution in dichloromethane (2 mL) was added drop-wise to a TFOH (0.385 g, 0.5 mmol, 1 eq) and (octyloxy)benzene (1.03 g, 5 mmol, 10 eq) mixture in dichloromethane (10 mL). This reaction mixture was stirred at room temprature (25 °C) under nitrogen until no starting material was detectable by TLC (approximately 10 min). Ethanol (10 mL) and water (10 mL) were successively added to quench the reaction. The phases were then separated, and the aqueous phase was extracted with dichloromethane. The combined dichloromethane layers were washed and dried (MgSO_4_). After removing the solvent, the remaining crude product was purified by silicon gel chromatography (petroleum ether) to yield a white product. The yield of 9,9′,9″-tris(4-(octyloxy)phenyl)-9,9′,9″-triphenyl-2,2′:7′,2″-terfluorene (TFO8) was 90%. MALDI-TOF-MS (*m*/*z*): calcd. For C_99_H_98_O_3_: 1334.8 [M^+^]; Found: 1334.1 (Figure S4). ^1^H NMR (400 MHz, CDCl_3_) δ 7.76 (d, *J* = 7.8 Hz, 6H), 7.55 (dd, *J* = 12.3, 5.2 Hz, 8H), 7.36 (ddd, *J* = 9.9, 8.5, 4.3 Hz, 4H), 7.30–7.18 (m, 21H), 7.17–7.11 (m, 7H), 6.79–6.72 (m, 6H), 3.89 (t, *J* = 6.5 Hz, 6H), 1.78–1.69 (m, 6H), 1.41 (d, *J* = 7.9 Hz, 6H), 1.35–1.22 (m, 30H), 0.90–0.85 (m, 10H). ^13^C NMR (101 MHz, CDCl_3_) δ 157.98, 157.95, 152.42, 152.16, 151.83, 146.19, 146.15, 140.85, 140.83, 139.68, 139.30, 138.89, 137.60, 137.57, 129.20, 128.31, 128.24, 128.18, 128.15, 127.68, 127.43, 126.74, 126.66, 126.61, 126.17, 124.74, 120.37, 120.32, 120.20, 114.19, 114.14, 67.91, 65.02, 64.94, 31.82, 29.37, 29.32, 29.25, 26.09, 22.67, 14.11. Elem. anal. calcd. For C_99_H_98_O_3_: C, 89.01; H, 7.39; O, 3.59. Found: C, 90.10; H, 7.56; O, 3.53 (Figure S5).

## 4. Conclusions

We carried out supramolecular approaches to clarify the origin of green emission bonds (g-bands) and discover the phenomenon of supramolecular luminescence in oligo/polyfluorenes and their PLEDs. An oligofluorenol-based, supramolecular polymer semiconductor with sterically hindered oligofluorenes was designed and synthesized via a Suzuki reaction. A variety of evidence for an aggregate g-band mechanism for oligofluorene-based, supramolecular conjugated oligomers was provided using solvent- or concentration-dependent nuclear magnetic resonance (NMR), dynamic light scattering (DLS) and photoluminescence spectra. The reversibility of the TFOH-1 g-bands in toluene via methanol titration, suggests that random supramolecular polymers or aggregates were generated by cooperative interchain hydrogen bonding and π–π stacking interactions. Therefore, our results strongly suggest that ketone defects are only a sufficient condition of g-bands, rather than a sufficient-necessary condition. Supramolecular electroluminescence will open a new door to organic devices in supramolecular optoelectronics.

## Supplementary Information
