# Supplementary material for: Supramolecular Luminescence from Oligofluorenol-Based Supramolecular Polymer Semiconductors
Source: Int J Mol Sci. 2013 Nov 13;14(11):22368–79. doi: 10.3390/ijms141122368 (PMC3856068; doi:10.3390/ijms141122368)
Supplement: Supplementary file 1 [file ijms-14-22368-s001.pdf]

Supplementary Information

Figure S1. ^{1}H NMR and ^{13}C NMR spectra of 9-phenyl-2-(4,4,5,5-tetramethyl-1,3, 2-dioxaborolan-2-yl)-fluoren-9-ol (TMB-PFOH).

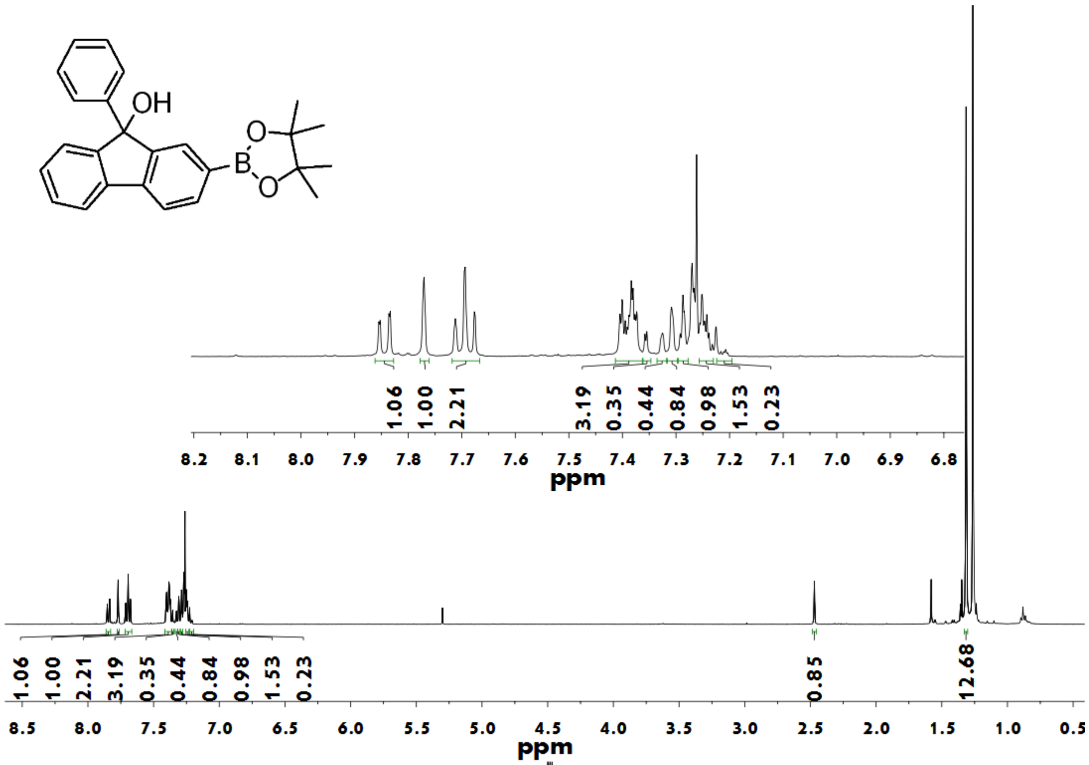

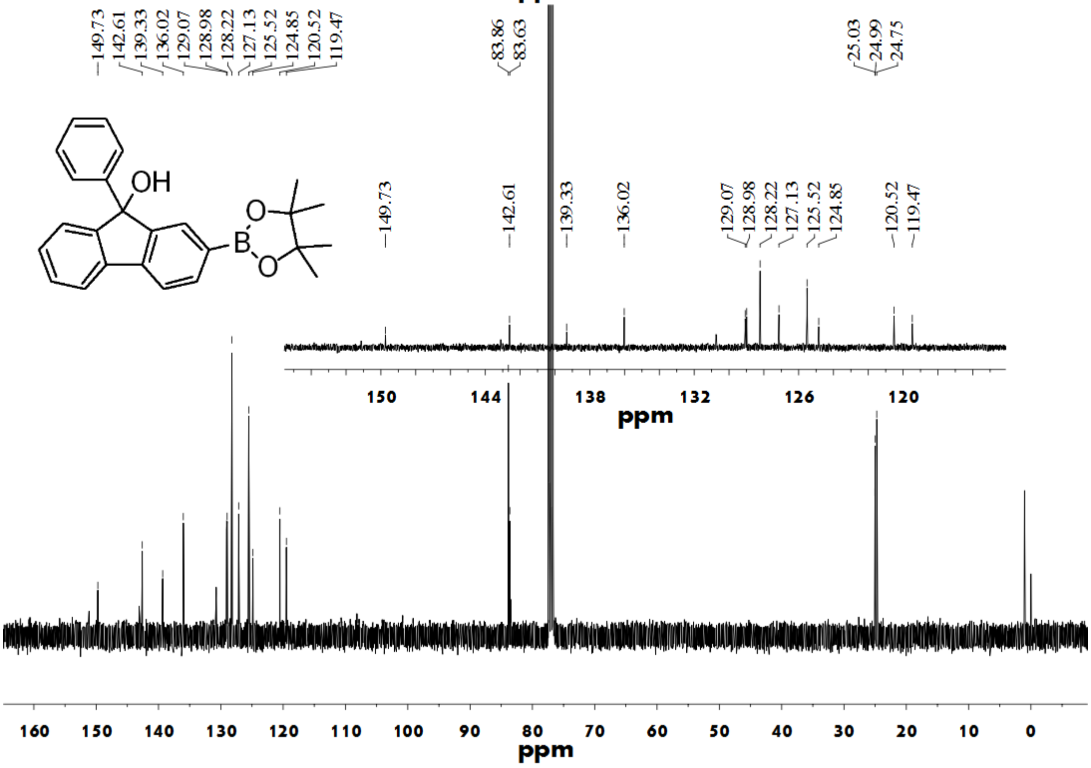

Figure S2. MALDI-TOF-MS spectra of 9'-(4-(octyloxy)phenyl)-9,9''-diphenyl-[2,2':7',2''-terfluorene]-9,9',9''-triol (TFOH).

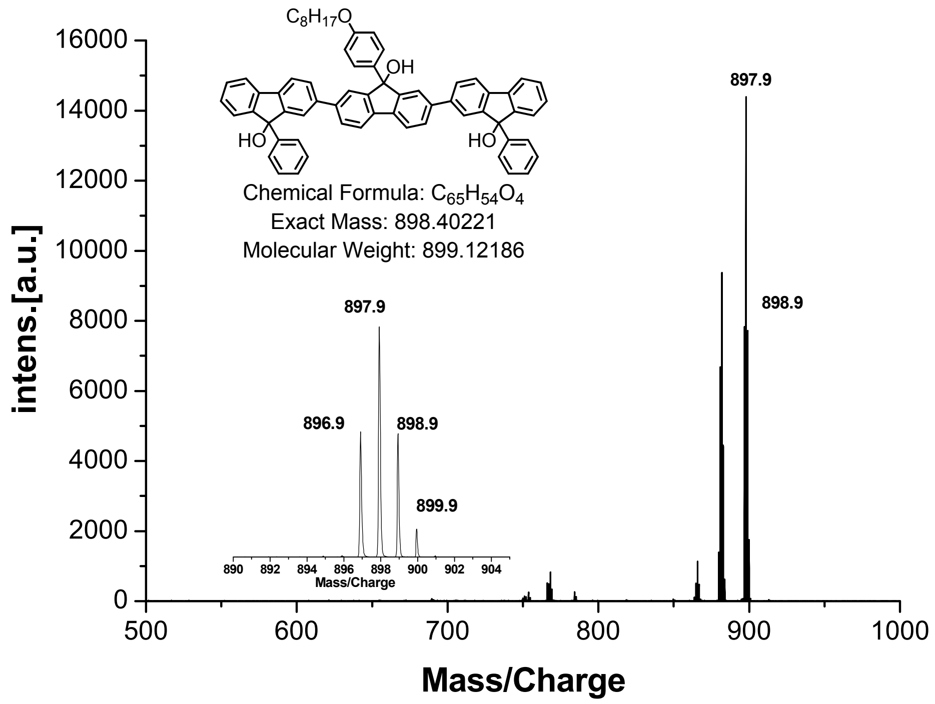

Figure S3. ^{1}H NMR and ^{13}C NMR spectra of 9'-(4-(octyloxy)phenyl)-9,9"-diphenyl-[2,2':7',2"-terfluorene]-9,9',9"-triol (TFOH).

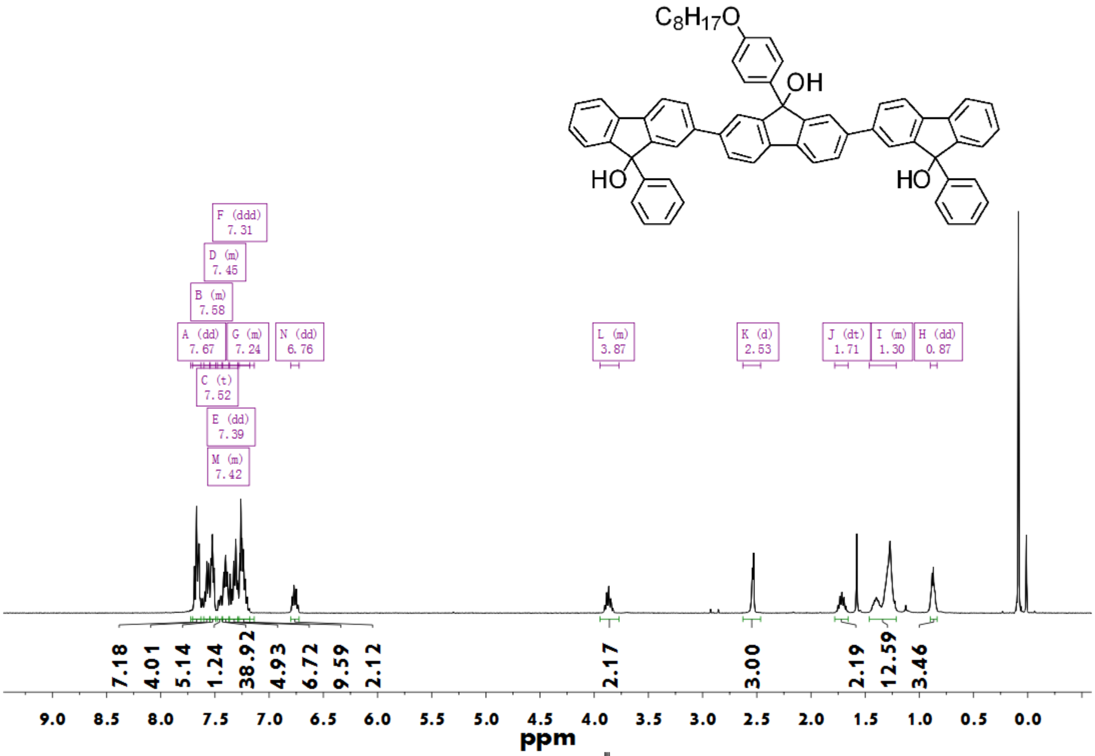

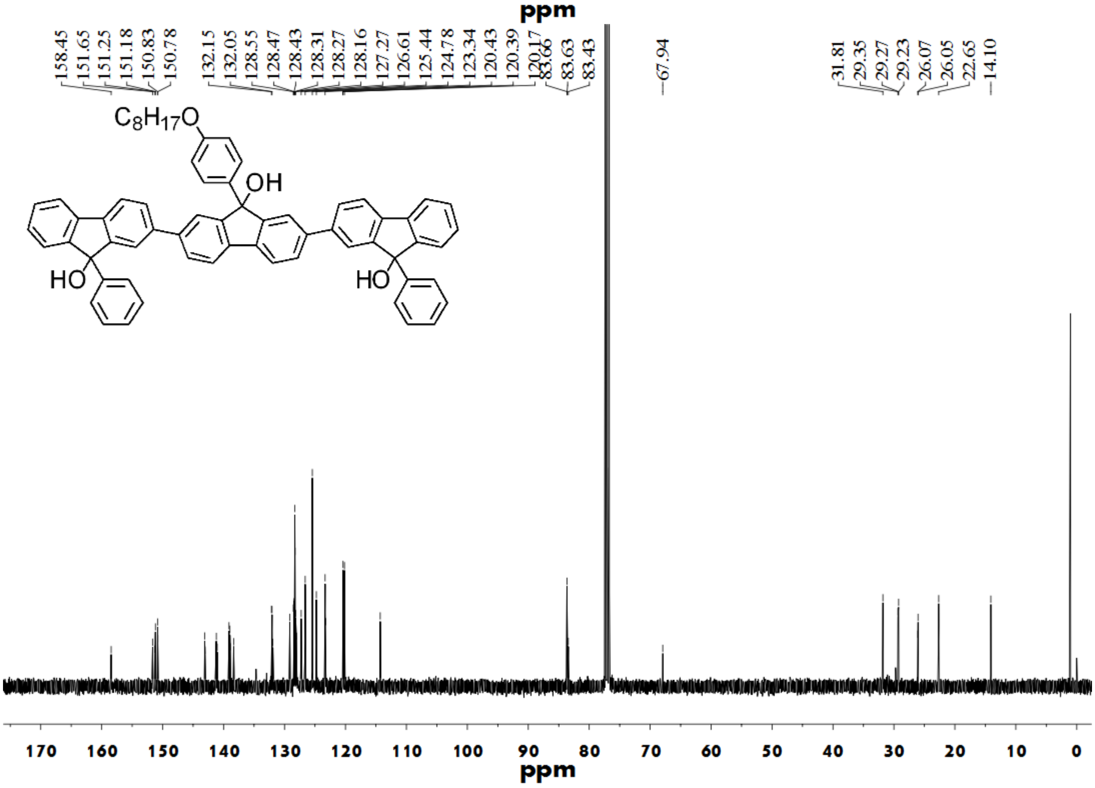

Figure S4. MALDI-TOF-MS spectra of 9,9',9''-tris(4-(octyloxy)phenyl)-9,9',9''-triphenyl-2,2':7',2''-terfluorene (TFO8).

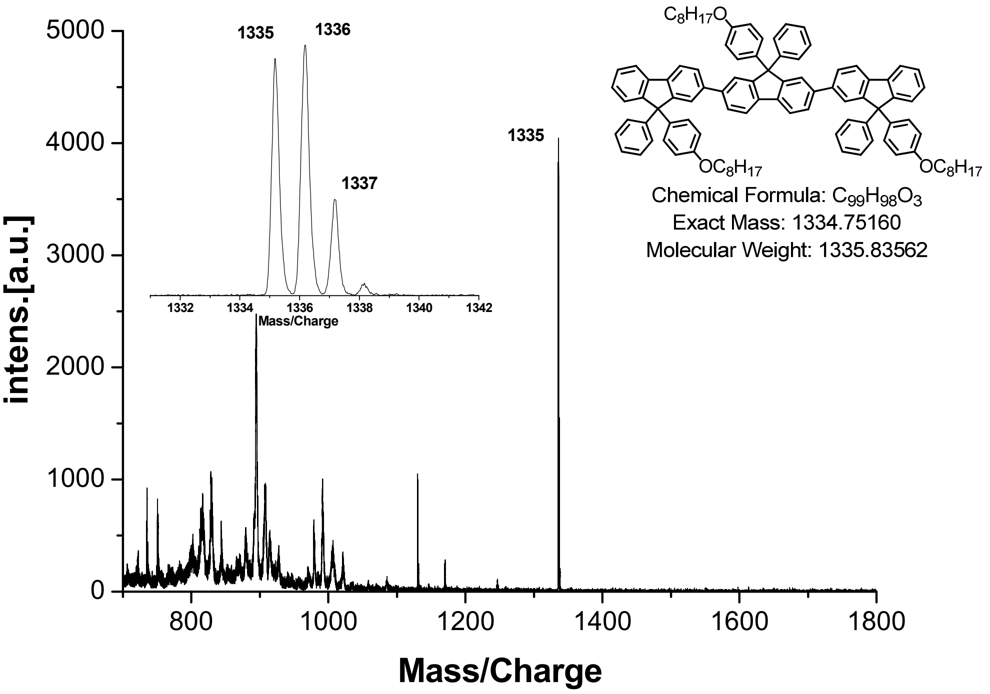

Figure S5. ^{1}H NMR and ^{13}C NMR spectra of 9,9',9''-tris(4-(octyloxy)phenyl)-9,9',9''-triphenyl-2,2':7',2''-terfluorene (TFO8).

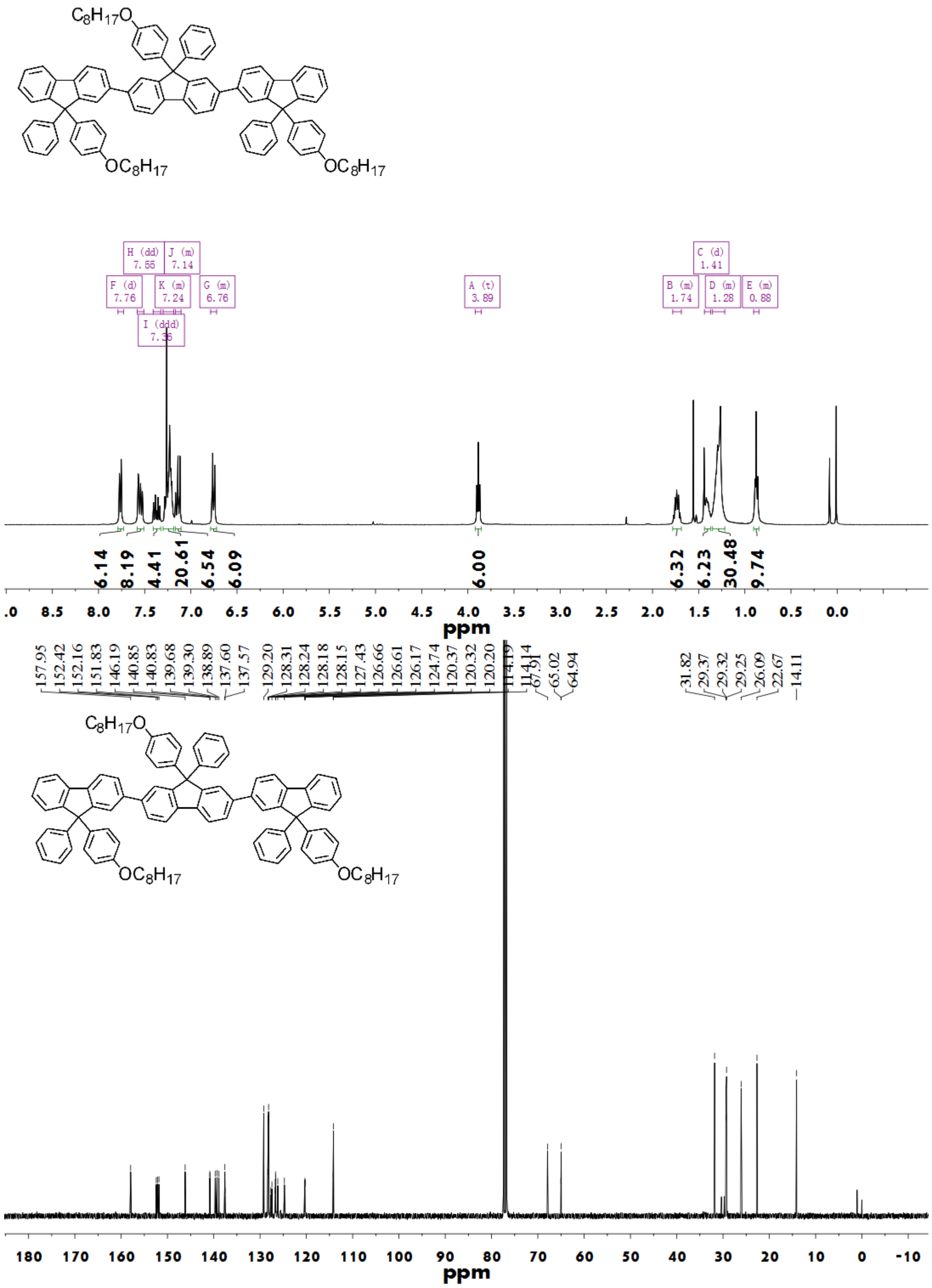

© 2013 by the authors; licensee MDPI, Basel, Switzerland. This article is an open access article distributed under the terms and conditions of the Creative Commons Attribution license (http://creativecommons.org/licenses/by/3.0/).
